# Plastome Characterization and Phylogenomic Analysis Yield New Insights into the Evolutionary Relationships among the Species of the Subgenus *Bryocles* (*Hosta*; Asparagaceae) in East Asia

**DOI:** 10.3390/plants10101980

**Published:** 2021-09-22

**Authors:** JiYoung Yang, Mi-Jung Choi, Seon-Hee Kim, Hyeok-Jae Choi, Seung-Chul Kim

**Affiliations:** 1 Research Institute for Ulleung-do & Dok-do, College of Natural Sciences, Kyungpook National University, Daegu 41566, Korea; whity@daum.net; 2 Department of Biology and Chemistry, Changwon National University, Changwon 51140, Korea; chlal1007@gmail.com; 3 Department of Biological Sciences, Sungkyunkwan University, Suwon 16419, Korea; desfilles@naver.com

**Keywords:** *Hosta*, Asparagaceae, RNA editing, mutation hotspots, positive selection, *Hosta capitata*

## Abstract

The genus *Hosta*, which has a native distribution in temperate East Asia and a number of species ranging from 23 to 40, represents a taxonomically important and ornamentally popular plant. Despite its taxonomic and horticultural importance, the genus *Hosta* has remained taxonomically challenging owing to insufficient diagnostic features, continuous morphological variation, and the process of hybridization and introgression, making species circumscription and phylogenetic inference difficult. In this study, we sequenced 11 accessions of *Hosta* plastomes, including members of three geographically defined subgenera, *Hosta*, *Bryocles*, and *Giboshi*, determined the characteristics of plastomes, and inferred their phylogenetic relationships. We found highly conserved plastomes among the three subgenera, identified several mutation hotspots that can be used as barcodes, and revealed the patterns of codon usage bias and RNA editing sites. Five positively selected plastome genes (*rbcL*, *rpoB*, *rpoC2, rpl16,* and *rpl20*) were identified. Phylogenetic analysis suggested (1) the earliest divergence of subg. *Hosta*, (2) non-monophyly of subg. *Bryocles* and its two sections (*Lamellatae* and *Stoloniferae*), (3) a sister relationship between *H. sieboldiana* (subg. *Giboshi*) and *H. ventricosa* (subg. *Bryocles*), and (4) reciprocally monophyletic and divergent lineages of *H. capitata* in Korea and Japan, requiring further studies of their taxonomic distinction.

## 1. Introduction

The genus *Hosta* Tratt. is one of the most important and popular landscaping and gardening plants throughout the northern temperate region. Hostas, or plantain lilies (Asparagaceae), have long been taxonomically and horticulturally investigated. With the estimates for the number of species ranging from 23 to 40, the genus is native to Northeast Asia, including Korea, Japan, China, and Russia [1,2,3,4,5,6]. The genus *Hosta* is believed to have originated in the bordering areas between the East China Sea and Sea/Sea of Japan, and Japan is considered the center of species diversity [2]. Three subgenera are commonly recognized based on their geographical distribution: *Hosta*, *Bryocles*, and *Giboshi* [2,5]. Owing to their showy flowers, splendid leaves, and shade tolerance, approximately 6300 cultivars have been developed through extensive hybridization and selection [7]. In addition to horticultural importance, some *Hosta* species have medicinal properties, including antioxidant and anti-inflammatory effects [8,9,10,11]. The wide range of *Hosta* species recognized in various taxonomic treatments is partly due to limited vegetative and reproductive characteristics and lack of comprehensive comparative analysis in East Asia, especially in Korea and Japan. Additionally, the lack of a robust overall phylogenetic framework within the genus hinders the evaluation of the degree of morphological variation and its taxonomic importance in species delimitation and phylogenetic relationships. Consequently, the boundaries between taxa of various ranks are still subject to debate. Nonetheless, approximately eight taxa (six species and two varieties; [5,12]) in Korea, 21–22 taxa (15 species and 7 varieties in Fujita [1]; 12 species and 9 varieties in Tamura and Fujita [6]) in Japan, and 4 species in China [3] have been subject to various infrageneric classification systems over several decades [1,2,4,13]. In particular, Schmid [2] proposed a new subg. *Giboshi*, which primarily includes species in Japan, whereas subg. *Bryocles* represents species mainly from Korea. The subg. *Hosta* consists of only one species endemic to China (*H. plantaginea*). With two additional new criteria applied to the taxonomic study of the genus, Zonneveld and Van Iren [4] maintained three subgenera of Schmid [2] but rearranged several taxa based on their genome size and pollen viability. One notable finding is that the species mainly belonging to the Korean subg. *Bryocles* tended to have a lower nuclear DNA content than the *Giboshi* subg. from Japan (17.2–19.3 pg vs. 21.3–26.5 pg) [4].

Of ten [2] and seven [4] sections recognized within two subgenera *Bryocles* and *Giboshi*, two sections, *Lamellatae* and *Stoloniferae* in subg. *Bryocles* are of particular interest, considering their main distribution in Korea. Schmid [2] placed four species, *H. venusta*, *H. minor*, *H. capitata*, and *H. nakaina,* in sect. *Lamellatae*, while placing *H. yingeri* and *H. laevigata* in *Arachnanthae* and *H. clausa* in *Stoloniferae*. By contrast, Zonneveld and Van Iren [4] placed *H. venusta*, *H. minor*, *H. jonesii*, *H. tibae*, and *H. tsushimensis* in sect. *Lamellatae* and three species, *H. clausa*, *H. capitata*, and *H. yingeri* in sect. *Stoloniferae*. One main difference is the placement of Korean endemic *H. jonesii* and two Japanese endemic species (*H. tibae* and *H. tsushimensis*) in sect. *Tardanthae* of subg. *Giboshi* [2] and sect. *Lamellatae* of subg. *Bryocles* [4]. The Korean *Hosta* has been a subject of intensive systematic and population genetic investigations during the late 1980s and the early 1990s [14,15,16,17,18,19,20,21,22]. These studies greatly improved our understanding of *Hosta* in Korea, providing baseline information about species relationships, evolutionary history, and population genetic structures.

Two recent phylogenomic studies provided additional insights into the phylogenetic relationships among species in Korea [23,24]. First, complete plastome sequences were assembled for representative Korean *Hosta* species [23]. While this study primarily focused on characterization and comparative plastome analyses, limited species relationships were inferred. For example, the maximum-likelihood (ML) tree suggests that *H. clausa* (sect. *Stoloniferae*) diverged first within the genus in Korea, followed by the divergence of two major lineages, including *H. capitata*-*H. ventricosa* (93% bootstrap support, BS) and the exclusively Korean endemics *H. venusta*-*H. minor* and *H. jonesii*-*H. yingeri* (100% BS). Neither Schmid’s [2] nor Zonneveld and Van Iren’s [4] classification system was supported by this study. However, it is necessary to interpret these results cautiously, given the limited sampling to address species relationships rigorously. A more recent and rigorous phylogenomic study was conducted based on 246 nuclear genes and whole plastome sequences using 55 accessions of Korean *Hosta* species, including numerous cultivars [24]. A few important findings of this study include (1) the trend of genome size reduction during the diversification of *Hosta* species in Korea, (2) cases of introgressive hybridization events between species (especially between *H. jonesii* and *H. yingeri* and very rarely between *H. jonesii* and *H. minor*), and (3) rapid diversification of Korean *Hosta* species during the late Miocene [24]. Although this study detected introgressive hybridization between species and inferred species relationships based on nuclear and chloroplast genomes, it was limited to assess overall phylogenetic relationships among species in three sections (*Bryocles*, *Stoloniferae*, and *Lamellatae*) of subg. *Bryocles* in East Asia. In particular, as pointed out in the study, phylogenetic relationships of two Japanese endemics, *H. tsushimensis* and *H. tibae*, relative to species in Korea, remain to be determined [24].

The advent of next-generation sequencing (NGS) technologies has enabled us to quickly assemble the complete plastome sequences of various plant lineages, providing opportunities to understand their molecular evolution and make inferences about phylogenetic relationships. As complementary to nuclear genes to document reticulate evolution and introgression, plastome phylogenomic studies have provided valuable information for resolving phylogenetic relationships at various taxonomic levels. Recently, complete plastome sequences have proven useful in understanding the evolutionary history of closely related species in East Asia (e.g., [25,26,27,28,29,30,31]). Furthermore, rapid accumulation of complete plastome sequences in the family Asparagaceae (55 plastome sequences; GenBank accessed 20 July 2021) serves as a reference, allowing us to reconstruct a robust phylogenetic framework among major lineages within the family as well as to gain insight into plastome evolution (e.g., [32]).

The aim of this study was to (1) assemble and characterize plastomes of additional infraspecific accessions of *Hosta* in Korea and three Japanese endemics (*H. sieboldiana, H. tibae*, and *H. tsushimensis*), (2) conduct a comprehensive comparative plastome analysis among three sections of subg. *Bryocles* and two other subgenera *Hosta* and *Giboshi*, (3) build reference plastome sequences for future phylogenomic analysis of the genus *Hosta*, and (4) determine phylogenetic relationships among species in subg. *Bryocles*. The answers to these questions should allow us to grasp plastome evolution and the fascinating evolutionary history of hostas in East Asia.

## 2. Results

### 2.1. Genome Size and Features

**Figure 1:**
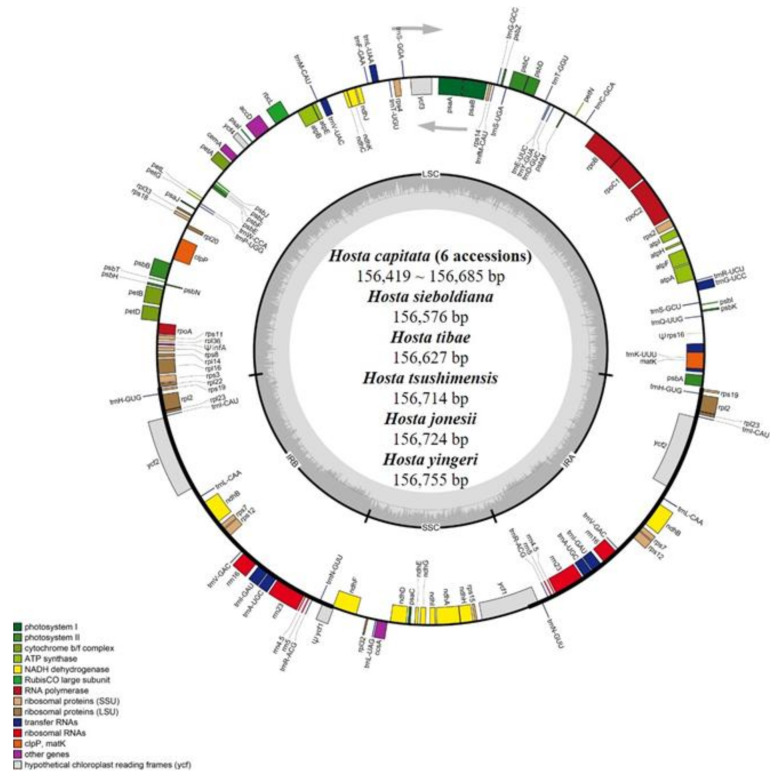
The 11 *Hosta* plastomes. The genes located outside the circle are transcribed clockwise, while those located inside are transcribed counterclockwise. The gray bar area in the inner circle denotes the genome’s guanine–cytosine (GC) content, whereas the lighter gray area indicates the genome’s adenosine–thymine (AT) content. LSC, SSC, and IR indicate large single-copy, small single-copy, and inverted repeats, respectively.

The plastomes of 11 newly sequenced *Hosta* accessions were 156,419 bp (*H. capitata*; Mt. BaekUn, Korea)–156,755 bp (*H. yingeri*) in length and comprised a large single-copy (LSC) region of 84,785–85,115 bp, a small single-copy (SSC) region of 18,202–18,232 bp, and two inverted repeat (IR) regions of 26,697–26,711 bp (Figure 1 and Table 1). Of the 11 plastomes of *Hosta*, *H. yingeri*, with a narrow distribution in the remote islands of the southwestern Korean Peninsula, had the largest cp genome (i.e., 156,755 bp) with the largest LSC region (i.e., 85,115 bp). The plastomes of *H. capitata* sampled from Korea were smaller than those from Japan: 156,419–156,423 bp and 156,576–156,685 bp, respectively (Table 1). No variation in the total number of genes (e.g., protein-coding, transfer RNA [tRNA], and ribosomal RNA [rRNA]) and IR size was observed among the six accessions of *H. capitata*.

The 11 newly sequenced accessions of *Hosta* contained 134 genes, including 85 protein-coding, eight rRNA, and 38 tRNA genes. And the overall guanine–cytosine (GC) content was slightly different ranging from 37.8 to 37.82% (Table 1). Additionally, 19 duplicated genes were present in the IR region (eight tRNA, four rRNA, and seven protein-coding genes). Fifteen genes (*atpF, ndhA, ndhB, petB, petD, rpl2, rpl16, rpoC1, rps12, trnA-UGC, trnG-*UCC, *trnI-*CAU, *trnK-*UUU, *trnL-*UAA, and *trnV-*UAC) contained a single intron, whereas *clpP* and *ycf3* contained two introns. The *infA* and *rps16* genes located in the LSC are pseudogenes. All 11 *Hosta* plastomes contained a partial *ycf1* gene of 1113 bp, located in the IRb/SSC junction region. However, the complete *ycf1* gene, which is located in the IR region at the SSC/IRa junction, was present in six *H. capitata* accessions with a length of 5403 bp and five other congeneric species (*H. jonesii, H. sieboldiana, H. tibae, H. tsushimensis*, and *H. yingeri*) with a length of 5412 bp.

**Figure 2:**
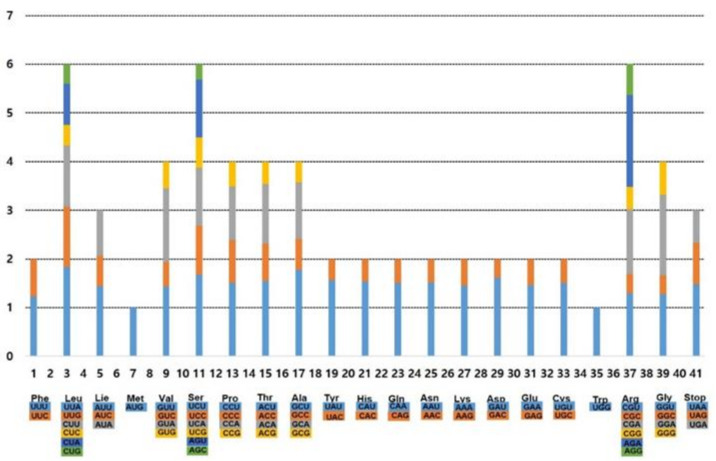
Codon distribution and relative synonymous codon usage in the 12 plastomes of *Hosta*.

The frequency of codon usage in 12 *Hosta* plastomes, including three subgenera, *Hosta*, *Bryocles*, and *Giboshi*, was calculated for the chloroplast genome based on the sequences of protein-coding and tRNA genes. The average codon usage in these species ranged from 23,096 (*H. plantaginea*; subg. *Hosta*) to 26,508 (*H. venusta*; subg. *Bryocles*; Supplementary Table S1). The average codon usage of the remainder was similar: 26,233 for *H. capitata* (Japan) and *H. jonesii* (subg. *Bryocles*); 26,492 for *H. clausa* and *H. minor* (subg. *Bryocles*); 26,275 for *H. capitata* (Korea, subg. *Bryocles*); 26,284 for *H. sieboldiana* (subg. *Giboshi*); 26,219 for *H. tibae* and *H. tsushimensis* (subg. *Bryocles*); and 26,215 for *H. yingeri* (subg. *Bryocles*). Moreover, we found that the distribution of codon types was consistent (Figure 2). In all 12 *Hosta* species in this study, identical chloroplast genes and codon usage patterns were detected (Supplementary Table S1). We found that codon usage was biased toward a high relative synonymous codon usage (RSCU) value for U and A at the third codon position.

The predicted number of RNA editing sites in 12 *Hosta* plastomes representing three subgenera was 67, with the same cut-off value of 0.8, and 24 of 35 protein-coding genes were predicted to undergo RNA editing (Supplementary Table S2). These genes included 12 photosynthesis-related genes (*atpA, atpF, ccsA, ndhA, ndhB, ndhD, ndhF, ndhG, petB, petD, psaB,* and *psbF*), eight self-replication genes (*rpl2, rpl20, rpoA, rpoB, rpoC1, rpoC2, rps8, and rps14*), and four other genes (*accD, clpP, matK*, and *ycf3*). We detected no RNA editing sites in 11 genes (i.e., *atpB, atpI, petG, petL, psaI, psbB, psbE, psbL, rpl23, rps2*, and *rps16*), which was identical in all 11 *Hosta* plastomes. Of the 12 *Hosta* plastomes included in this study, RNA editing sites in *petD* (four sites; all from leucine to phenylalanine) and *psaB* (one site; from threonine and methionine) were found uniquely in *H. capitata* (Iya Valley accession, Japan) and *H. plantaginea*, respectively. The *ndhB* gene showed the highest number of potential editing sites (15 sites), followed by *rpoB* (six sites), *ndhF* (five sites), and *ndhD* (four sites). *Hosta clausa* contained only two RNA editing sites in *ndhD*, while the other *Hosta* species had four RNA editing sites. In the case of *H. minor*, four RNA editing sites in the *clpP* gene were found, while the other *Hosta* species had only one site.

Based on the K2P model, we calculated the pairwise sequence distances among six accessions of *H. capitata* (subg. *Bryocles*, sect. *Stoloniferae*) distributed disjunctly in Korea and Japan (Supplementary Table S3). The distance between the two accessions of *H. capitata* from Korea was 0.00003, while it was 0.00012 on average (±0.00000256, standard deviation, SD) for four accessions from Japan. The average pairwise distance between accessions in Korea and Japan was 0.000198 ± 0.00002659. In terms of the K2P distance for a sister species identified in this study, we found 0.00026 between *H. jonesii* and *H. tsushimensis* (subg. *Bryocles*, sect. *Lamellatae*) and 0.00008 between *H. ventricosa* (subg. *Bryocles*, sect. *Bryocles*) and *H. sieboldiana* (subg. *Giboshi*). The distance between the two Korean endemics, *H. minor* and *H. venusta*, was 0.00003 ± 0.0000173.

### 2.2. Comparative Plastome Analyses

**Figure 3:**
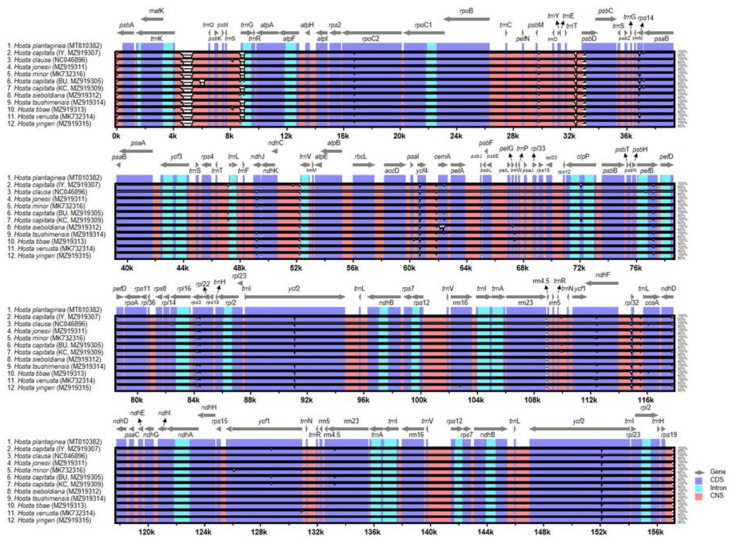
Visualization of the alignment of 12 *Hosta* species chloroplast genome sequences. The VISTA-based identity plots using mVISTA in the Shuffle-LAGAN show the sequence identity of 12 *Hosta* species. The vertical scale indicates the percent identity from 50 to 100%. Coding and non-coding regions are in blue and pink, respectively. Gray arrows above the alignment indicate the position and direction of each gene.

**Figure 4:**
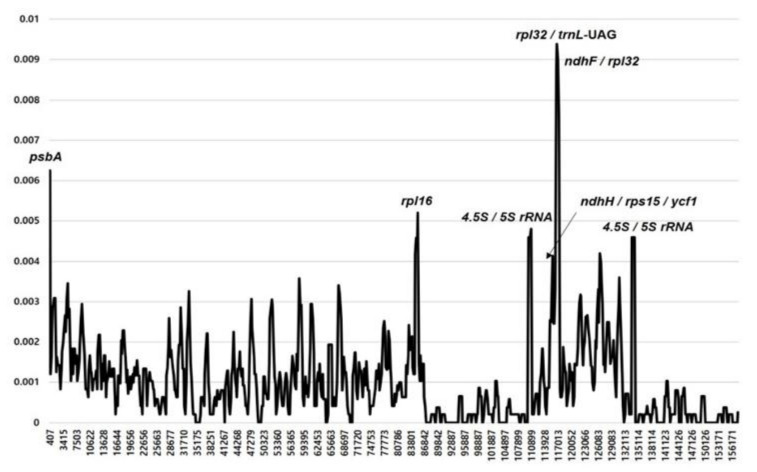
Sliding window analysis of the 12 whole chloroplast genomes of *Hosta* species. X-axis: position of the window midpoint, Y-axis: nucleotide diversity within each window.

The plastomes of 11 *Hosta* species, including the subg. *Giboshi* (*H. sieboldiana*) and subg. *Bryocles* (three accessions of *H. capitata*, *H. jonesii*, *H. minor*, *H. tibae*, *H. tsushimensis*, *H. venusta*, and *H. yingeri*), were plotted using mVISTA and the annotated *H. plantaginea* (subg. *Hosta*) plastomes as a reference (Figure 3). The results indicated that the LSC region was the most divergent, and the two IR regions were highly conserved. Additionally, the non-coding regions were more divergent and variable than the coding regions. Sliding window analysis using the DnaSP program revealed highly variable regions in the plastomes of 12 *Hosta* species representing three subgenera (Figure 4). Comparison of the 12 plastomes revealed that the average value of nucleotide diversity (π) over the entire chloroplast genome was 0.00207, with the most variable region (π = 0.00939) being the *rpl32*/*trnL*-UAG intergenic region. We also detected two additional highly variable regions, one genic and the other intergenic, *psbA* (π = 0.00625) and *ndhF*/*rpl32* (π = 0.00877). Three other variable regions (>π = 0.004) were *rpl16* (π = 0.00521), 4.5S rRNA/5S rRNA (π = 0.00481), and *ndhK*/*rps15*/*ycf1* (π = 0.00413).

Positive selection analysis performed using the EasyCodeML [33] program with the site-specific model based on CODEML algorithms [34] allowed us to identify positively selected genes among 12 *Hosta* plastomes (Table 2). Among the conserved genes, five genes with positively selected sites were identified with statistically significant LRT *p* values (<0.05) (Table 2). These genes included two ribosomal protein large subunit genes (*rpl16* and *rpl20*), two subunits of RNA polymerase (*rpoC2* and *rpoB*), and one large subunit of rubisco (*rbcL*), based on the M7 and M8 model. All five genes had one to two positive sites: *rpoC2*, *rpoB*, and *rbcL* (one site each), *rpl16* (two sites), and *rpl20* (two sites). However, all but five genes (70 out of 75 chloroplast genes) had an average Ka/Ks ratio below 1, indicating that these genes were subjected to strong purifying selection in the *Hosta* chloroplast.

### 2.3. Plastome Phylogenomic Analysis of Hosta in East Asia

**Figure 5:**
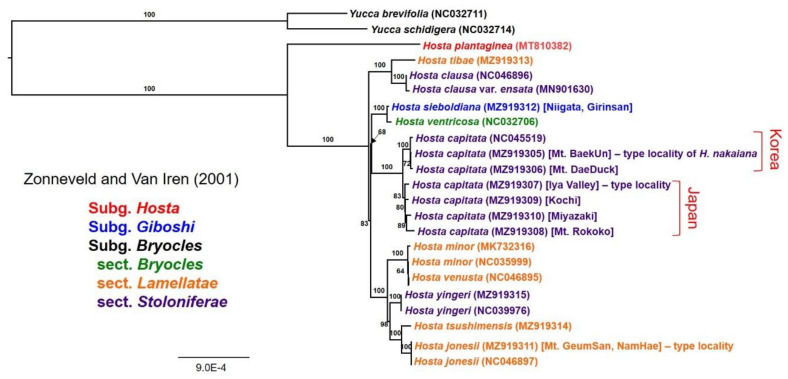
The maximum-likelihood (ML) tree using IQ-TREE inferred from 21 *Hosta* accessions and two *Yucca* species. The complete plastomes of 21 *Hosta* accessions are labeled in red (subg. *Hosta*), blue (subg. *Giboshi*), green (subg. *Bryocles*, sect. *Bryocles*), orange (subg. *Bryocles*, sect. *Lamellatae*), and purple (subg. *Bryocles*, sect. *Stoloniferae*). The bootstrap value based on 1000 replicates is shown on each node. Two *Yucca* species were used as an outgroup, and columns on the right indicate the two habitats of Japan and Korea.

Based on a total of 159,206 aligned nucleotide sites and 1383 parsimony informative sites, an ML analysis (“K3Pu+F+I” as the best-fit model) was performed to infer phylogenetic relationships among 21 plastome accessions of *Hosta* species, including subg. *Hosta* (one species, *H. plantaginea*), subg. *Giboshi* (one species; *H. sieboldiana*), and subg. *Bryocles* (nine species: *H. tibae*, *H. clausa*, *H. minor*, *H. venusta*, *H. yingeri*, *H. tsushimensis*, *H. jonesii*, *H. ventricosa*, and *H. capitata*) (Figure 5). Two *Yucca* species plastomes from the asparagus family were used as outgroups, and the ML tree strongly supported the monophyly of the genus *Hosta* (100% BS). The ML tree showed that the subg. *Hosta* diverged first within the genus *Hosta,* followed by major radiation of two subgenera, *Bryocles* and *Giboshi,* in Korea and Japan. A sole representative of subg. *Giboshi* (*H. sieboldiana*) from Japan was embedded in the subg. *Bryocles* and was sister to *H. ventricosa* (sect. *Bryocles*), which is endemic to China. Two other sections of subg. *Bryocles*, namely *Lamellatae* and *Stoloniferae*, were not monophyletic. Four major lineages based on plastomes were identified with strong support (100% BS): (1) the clade of *H. tibae* (sect. *Lamellatae*) and *H. clausa* (sect. *Stoloniferae*), (2) the clades of *H. minor*, *H. venusta*, *H. tsushimensis*, and *H. jonesii* (sect. *Lamellatae*) and *H. yingeri* (sect. *Stoloniferae*), (3) the clade of *H. sieboldiana* (subg. *Giboshi*) and *H. ventricosa* (sect. *Bryocles*), and (4) the *H. capitata* clade (Figure 5). The phylogenetic relationships among these four lineages were moderately supported (68% and 83% BS). Of the six accessions of *H. capitata* sampled from Korea and Japan, the ML tree indicated Korean accessions (NC045519, Mt. DaeDuck and Mt. BaekUn) and Japanese accessions (Kochi, Miyazaki, Mt. Rokko, and the Iya Valley) were reciprocally monophyletic.

## 3. Discussion

### 3.1. Plastome Evolution in Subgenus Bryocles

For the first time, we generated the complete chloroplast genome of *Hosta* species in Japan, *H. sieboldiana* (subg. *Giboshi*) and two species of subg. *Bryocles*, sect. *Lamalletae* (*H. tibae* and *H. tsushimensis*) and compared them to those of the major members of subg. *Bryocles* (three sections: *Bryocles*, *Lamalletae*, and *Stoloniferae*) and subg. *Hosta* (*H. plantaginea*). As expected, the plastomes of *Hosta* species representing three subgenera were highly conserved, with a total length ranging from 156,419 to 156,755 bp, and all contained 37.8–37.82% GC content and showed no structural variation or gene content rearrangements (Table 1). The highly conserved nature of plastomes among congeneric species is consistent with that of other angiosperms in East Asia (e.g., [26,28,29,30,35,36,37]). Despite the conserved nature of the 11 *Hosta* plastomes generated in this study, they will be highly valuable and of great importance as references for additional *Hosta* species, especially those from Japan (subg. *Giboshi*), sequenced for future plastome phylogenomic studies.

To understand the molecular evolution and environmental adaptation in the genus *Hosta*, the codon usage bias and RNA editing sites were examined to balance mutational bias and natural selective forces [38,39,40]. All 12 *Hosta* species showed identical codon usage patterns and were biased toward high RSCU values of U and A at the third codon position. A similar phenomenon has been reported in other angiosperms [41] and algal lineages [38]. The 12 photosynthesis-related genes, eight self-replication genes, and four other functional genes (*accD*, *clpP*, *matK*, and *ycf3*) were predicted to undergo RNA editing. The *ndhB* gene had the highest number of potential editing sites (15), followed by *rpoB* (6 sites), *ndhF* (5 sites), and *ndhD* (4 sites) in all 12 *Hosta* plastomes. The number of editing sites in those genes agrees with the results of previous studies (e.g., [30,42,43,44]). In general, the most frequent RNA editing sites were found in *ndhB* and *ndhD* genes, but 12 *Hosta* species showed that the *rpoB* gene also had a high number of potential editing sites.

The identification of mutation hotspots or highly variable regions of plastomes can be highly valuable for unraveling the complex maternal evolutionary history of *Hosta* and for developing DNA barcoding markers for phylogeographic and horticultural applications. We found that the most variable region among the 12 *Hosta* plastomes representing three subgenera was the *rpl32*/*trnL*-UAG intergenic region (π = 0.00939), followed by one intergenic region (*ndhF*/*rpl32*, π = 0.00877) and one genic region (*psbA*, π = 0.00625). Additionally, five other variable regions (> π = 0.004), including *rpl16*, *ndhK*/*rps15*/*ycf1*, and 4.5S rRNA/5S rRNA, were identified as useful alternative markers. A previous study [23] based on six Korean *Hosta* plastomes identified one tRNA (*trnL*-UAG, π = 0.012; the same region identified as *rpl32*/*trnL*-UAG in this study), two protein-coding genes (*psbA*, π = 0.010; *ndhD*, π = 0.012), and one intergenic region (*ndhF*/*rpl32*, π = 0.12 as reported by Lee et al. [23] but the correct value should be 0.012). We included plastome accessions from two additional subgenera (*Hosta* and *Giboshi*) and identified the same three hotspot regions, including *rpl32*/*trnL*, *ndhF*/*rpl32*, and *psbA*. Therefore, these three regions should be highly valuable for future DNA barcoding and phylogeographic studies. As expected, most hotspot regions were detected in intergenic regions in LSC and SSC, while IR regions maintained a high homogeneity and conserved nature of the rRNA gene [45,46]. A relatively high Pi (π) value (0.00481) of *Hosta* plastomes was detected in the intergenic region between 4.5S rRNA and 5S rRNA from the IR region, and similar results showing high variability in the IR region, that is, *rps7*/*ycf68*, were found in *Dasypogon bromoliifolius* (Dasypogonaceae, subclass Commelinidae) [47]. We identified one divergent intergenic region from IRs in *Hosta* species in East Asia, which can be valuable barcodes for resolving infrageneric relationships.

Most plastome genes evolved under purifying selection due to functional constraints throughout the chloroplast genome evolution [48,49,50,51]. In 12 plastomes of the genus *Hosta*, we found that all but five genes (70 out of 75 genes) were under strong purifying selection with an average Ka/Ks ratio of <1. The selection pressure of protein-coding genes in the genus *Hosta* affected the major functional genes *rbcL*, *rpoB*, *rpoC2*, *rpl16*, and *rpl20* in chloroplasts. Several studies have previously reported the positive selection of the major functional genes throughout plastome evolution (e.g., [50,51,52,53]). Furthermore, the essential gene of a modulator of the photosynthetic *rbcL* gene under positive selection has been reported in eudicot lineages, such as *Fragaria* (Rosaceae, [49]), *Rubus* (Rosaceae [44]), *Gossypium* (Malvaceae [54]), *Panax* (Araliaceae [50]), and monocot lineage Poaceae grass after the C3-C4 photosynthetic transition [52]. The positive selection of functional genes during plastome evolution is related to adaptation to environmental changes such as temperature, drought, carbon dioxide concentration, photosynthetic rate, and ecological niche or coevolutionary processes [52,55]. Therefore, we suggest that the selection patterns detected among *Hosta* species could be associated with adaptation to climate change in East Asia, especially in Korea and Japan, during the Miocene when rapid diversification of *Hosta* species occurred [24].

### 3.2. Phylogenetic Relationships among Species of the Bryocles Subgenus in East Asia

One of the main objectives of this study was to determine phylogenetic relationships among species of *Hosta* in three sections (*Bryocles*, *Lamellatae*, and *Stoloniferae*) of subg. *Bryocles* [4]. While previous studies assessed these phylogenetic relationships based on molecular markers [23,24], limited geographical sampling or incomplete species coverage hindered our full understanding of overall species relationships. Based on an infraspecific sampling of *H. capitata* from Korea and Japan and additional accessions of Korean endemics (*H. jonesii* and *H. yingeri*) and two Japanese endemics (*H. tsushimensis* and *H. tibae*), we were able to assess the phylogenetic relationships among species within the subg. *Bryocles*. With the caveat that plastome phylogenomics can be subject to hybridization, introgression, and incomplete coverage of subg. *Giboshi*, we cautiously interpreted our results based on the most current infrageneric classification system of Zonneveld and Van Iren [4], which corresponds well with the eight sections of Fujita [1]. First, in terms of intersubgeneric relationships, subg. *Hosta*, which includes only one species (*H. plantaginea*) from China, diverged first, followed by major speciation of two subgenera *Bryocles* in Korea and *Giboshi* in Japan. The early divergence of subg. *Hosta* and its genomic distinctions were further confirmed in previous studies [4,24,56]. Furthermore, its subgeneric rank based on several autapomorphies (e.g., nocturnal, white, and fragrant flowers, large genome size) seems to be well justified [1,13].

The subg. *Giboshi* (*H. sieboldiana*) was deeply embedded within subg. *Bryocles*, making it paraphyletic. A much broader sampling of the subg. *Giboshi* from Japan is mandatory to further test the monophyly of *Giboshi* and phylogenetic relationships among species within the subgenus. Nevertheless, the current plastome phylogenomic analysis suggests that geographical distribution may not be a good characteristic for defining subgeneric ranks within the genus, requiring further confirmation in later studies. In the sectional ranks within the subg. *Bryocles*, neither *Lamellatae* nor *Stoloniferae* are monophyletic. Three distinct lineages within the sect. *Stoloniferae*, *H. clausa*, *H. yingeri*, and *H. capitata* were closely related to either sect. *Lamellatae* or a clade containing subg. *Giboshi* and sect. *Bryocles*. The phylogenetic position of *H. yingeri* based on the plastome sequences is consistent with previous reports; *H. yingeri* is sister to the clade containing *H. jonesii* [23,24]). *Hosta yingeri* shows unique floral characteristics (i.e., a funnel-shaped perianth, unequal stamens with three long and three short, and a persistent bract at flowering) and is endemic to isolated continental islands in southwest Korea [12]. As suggested by the incongruence between 246 single- and low-copy nuclear genes and whole plastome sequences, it is likely that these two species (*H. jonesii* and *H. yingeri*) shared a maternal parent or that they experienced hybridization [24]. The earliest divergence based on nuclear genes, unique floral features, and crossing experiments with its congeneric species (<60% pollen viability with other Korean endemics) suggest a distinct lineage within Korea, and further studies including species from Japan (subg. *Giboshi*) may shed new light on the origin and evolution of this unique taxon in Korea.

For the first time, we determined the phylogenetic relationships of two Japanese endemics, *H. tsushimensis* and *H. tibae*, based on complete plastome sequences. Despite their placement within the same section (sect. *Tardanthae* in Fujita [1] and Schmid [2]; sect. *Lamellatae* in Zonneveld and Van Iren [4]), our study strongly suggests that *H. tsushimensis* and *H. tibae* may not be closely related to each other, at least based on their plastomes. *Hosta tsushimensis* is endemic to the island of Tsushima, which lies between the Korean Peninsula and the Japanese Archipelago, and is known to be closely related to *H. minor*, *H. venusta*, and *H. jonesii* [22]. Of several morphologically related species, this study strongly suggests that *H. jonesii* is the most closely related to *H. tsushimensis* (100% BS). These two species occur in the Korea Strait, connecting the East Sea and the Sea of Japan and the East China Sea, and also share similar DNA content, further strengthening their close relationship: 17.5 ± 0.09 pg for *H. jonesii* and 17.3 ± 0.12 pg for *H. tsushimensis* [4]. Allozymes also suggested that they were most closely related to each other [21]. Based on population-level sampling, it is yet to be determined whether *H. tsushimensis* migrated from Korea to Tsushima Island during the Pleistocene and speciated on the island [57]. Alternatively, Chung [15] argued for the origin of *H. tsushimensis* from Korean endemic *H. minor* elements, which requires future studies. When *H. yingeri* (19.1 ± 0.06 pg) is excluded from the clade containing *H. minor*-*H. venusta*-*H. tsushimensis*-*H. jonesii* owing to chloroplast capture [24], the species in this clade have a small genome size, narrowly ranging from 17.2 ± 0.23 pg for *H. minor* to 17.5 ± 0.09 pg for *H. jonesii* [4]. In this study, the phylogenetic position of *H. tibae* was not expected, considering its morphological similarity to *H. minor*/*H. venusta*, *H. jonesii*, and *H. tsushimensis* [2]. *Hosta tibae* is considered a variety of *H. tsushimensis* [58]. *Hosta tibae* occurs narrowly in a small mountainous area around Nagasaki (Kyushu) with a flowering period in late September, while *H. tsushimensis* occurs on Tsushima Island and exhibits a flowering period from August to early September. The discovery of intermediate *H. tibae* populations from the mid-elevation of Inasa-dake in Nagasaki City, Kyushu, with intermediate morphology and phenology with *H. tsushimensis*, was the basis for the varietal rank recognition of *H. tibae*. However, our study strongly suggests that *H. tibae* shares its most recent plastome common ancestor with *H. clausa* in sect. *Stoloniferae* (100% BS) The DNA content between the two species is quite different, i.e., *H. tibae* (17.6 ± 0.03 pg) and *H. clausa* (19.2 ± 0.18 pg), but whether this close relationship is due to chloroplast capture, shown in the clade of *H. yingeri* (19.1 ± 0.06 pg) and *H. jonesii* (17.5 ± 0.09 pg) is uncertain. If this relationship is further confirmed based on nuclear markers, then morphological similarities between *H. tibae* and *H. tsushimensis*-*H. jonesii*-*H. minor* can be explained by convergence or parallelism. *Hosta clausa*, including var. *ensata* (= *H. ensata*), occurs in central and northern Korea and northeastern China (southern Jilin and southern Liaoning). This species is morphologically, palynologically, and isozymatically distinct from other Korean species [15,59]. Without nuclear gene phylogenies, it seems difficult to determine whether this species’ inference truly reflects their evolutionary history or is because of chloroplast capture between central/northern Korea endemic *H. clausa* and the southern elements of *H. tsushimensis*-*H. jonesii*-*H. tibae* during 11.5 Mya and 3.75 Mya [24].

The phylogenetic position of *H. ventricosa* in sect. *Bryocles* is elusive. The only natural tetraploid (2n = 4X = 120) *H. ventricosa* with known pseudogamous apomixis is the sole member of sect. *Bryocles* and is native to southeast and south-central China. Pollen morphology [59] and the genome size (19.6 ± 0.10 pg for *H. ventricosa* and 19.2 ± 0.18 pg for *H. clausa* [4]) seem to suggest its close relationship to *H. clausa*, which is morphologically not very similar. The similarity of chromosome shapes between *H. clausa* and *H. ventricosa* was also suggestive of sharing a common ancestor [4,60]. However, this study strongly suggests that the Chinese endemic *H. ventricosa* subg. *Bryocles* may be closely related to the Japanese elements of hostas, such as *H. sieboldiana* in subg. *Giboshi* (100% BS). However, the genome size comparison between the two species may not suggest their close relationships: 23.6 ± 0.36 pg and 19.6 ± 0.10 pg for *H. sieboldiana* and *H. ventricosa*, respectively. Considering the unique features of *H. ventricosa* (e.g., distinctly urn-shaped perianth, rugulate pollen, apomictic, and tetraploid), it is difficult to determine whether *H. ventricosa* is closely related to subg. *Giboshi* in Japan or any of the three distinct Korean lineages, namely, *H. capitata*, *H. clausa*, or *H. yingeri*. Based on the complete plastome sequences, *H. ventricosa* is more closely related to *H. capitata* than to *H. clausa* or *H. yingeri*. Yoo et al. [24] showed that *H. ventricosa* shares its most recent common ancestor with *H. clausa* based on nuclear genes, while it is closely related to *H. capitata* based on plastome sequences. Excluding the species sampled in our study (i.e., *H. tibae*, *H. tsushimensis*, and *H. sieboldiana*), the inferred phylogenetic relationships for the remaining species are congruent with that reported by Yoo et al. [24], perhaps suggesting that taxonomic sampling affected plastome relationships. To sort out taxonomic sampling, hidden paralogy, or incomplete lineage sorting as causes of phylogenetic incongruences, it is necessary to have broader sampling, especially of species from Japan, and conduct genome-wide analysis based on nuclear and chloroplast genomes.

This study also provides insights into the degree of variation in plastomes within *H. capitata* and its taxonomic relationships in Korea and Japan. *Hosta capitata* occurs mainly in central and southern Korea and Japan (Shikoku, Kyushu, and Honshu). The closely related yet taxonomically elusive *Hosta nakaiana* occurs primarily in Japan (Kyushu and Honshu) and in one southern province in Korea (Mt. BaekUn, Jeollanam-do). Interestingly, *H. capitata*, which occurs primarily in Korea, has a type locality in Japan (Iya Valley, Tokushima Prefecture), whereas *H. nakaiana*, mainly known in Japan, has a type locality in Korea (Mt. BaekUn, Jeollanam-do Province) [2]. While Fujita [1], Chung [15], Zonneveld and Van Iren [4], and Tamura and Fujita [6] considered *H. capitata* and *H. nakaiana* conspecifics, specific taxonomic treatments have also been proposed [2,13]. For the first time, we included populations of *H. capitata* sensu lato from Korea and Japan, and the whole plastome sequences suggested not only the monophyly of *H. capitata* (100% BS) but also two reciprocally monophyletic lineages, one Korean (100% BS) and the other Japanese (88% BS). The monophyly of *H. capitata* was also supported by nuclear and chloroplast genomes [24]. The sister relationship between *H. capitata* and the clade containing *H. sieboldiana* and *H. ventricosa* in this study was rather moderate or weak (68% BS). It appears that conspecific populations of *H. capitata* sampled in Korea and Japan are genetically as divergent as shown in other interspecific comparisons; intraspecific divergence in *H. capitata* (0.000198) is comparable to the interspecific comparison between *H. jonesii* and *H. tsushimensis* (subg. *Bryocles*) and significantly greater than that of the intersubgeneric *H. ventricosa* (subg. *Bryocles*)-*H. sieboldiana* (subg. *Giboshi*) (0.00008) and intrasubgeneric *H. minor*-*H. venusta* (subg. *Bryocles*) (0.00003) comparisons. Whether this genetic divergence in plastomes is also reflected in their morphological differentiation in the two regions is yet to be determined. Additionally, the taxonomic distinction between *H. nakaiana* and *H. capitata*, which may reflect the genetic divergence between the two regions, should be investigated thoroughly based on phylogenomic and phylogeographic studies.

Lastly, this study further confirmed the relationship between *H. minor* and *H. venusta* [23,24]. *Hosta minor* is a Korean endemic that occurs in southern and eastern Korea, while *H. venusta* is endemic to Jeju Island. A previous study suggested that the Jeju Island endemic *H. venusta* accessions were embedded within the *H. minor* clade, with little genetic differentiation between them [24]. Similar levels of nucleotide diversity (π = 0.004%) existed between the two species despite different sample sizes (12 accessions for *H. minor* and six accessions for *H. venusta*). Significantly lower genetic variation within each species than with other congeneric species (e.g., 0.049 for *H. clausa*, 0.044 for *H. capitata*, and 0.031 for *H. jonesii*) may have contributed to the low genetic differentiation between *H. minor* and *H. venusta*. Although Yoo et al. [24] argued that *H. minor* and *H. venusta* occur on Jeju Island and altitudinal clinal variation can be seen in *H. venusta* on Mt. Halla, we are uncertain about the taxonomic identity of *H. minor* on Jeju Island. Previous studies by Chung et al. identified the hostas on Jeju Island as *H. venusta* [14,15,16,19,20,21,22,59], and several floristic treatments and monographic studies have recognized *H. venusta* only on the island [5,12]. While *H. venusta* accessions failed to form a clade and instead were nested in the *H. minor* clade, taxonomic identities of certain accessions sampled on Jeju Island require further confirmation, for example, *H. minor* f. *alba* (15,261 and 15,263) and *H. minor* (15,257) (Figure 2 of Yoo et al. [24]). As these accessions and some cultivated ones are excluded, it appears that *H. venusta* forms a monophyletic group in nuclear phylogeny, but is deeply embedded within *H. minor*, a progenitor species in southern Korea [24]. In the founder effect model, as a geographically local speciation model, a monophyletic island daughter species nested within a paraphyletic continental progenitor species can be expected before reaching reciprocally monophyletic progenitor and derivative species pairs [61]. Any taxonomic treatment at the species level requires further detailed morphological and phylogeographic studies based on a broader population-level sampling of the two species.

## 4. Materials and Methods

### 4.1. Plant Materials

This study sampled six accessions of *H. capitata* from Korea and Japan (Supplementary Table S4). Unlike Schmid’s [2] and Maekawa’s [13] views, we took a broad view of *H. capitata*: *H. nakaiana*, which is native to Japan and Korea and is considered a synonym of *H. capitata*, which occurs in Korea and southwestern Japan [15]. In Korea, one accession of *H. capitata* was sampled from Daeduck Mountain (Gangwon-do Province), representing the northernmost population, while the other was sampled from BaekUn Mountain (Jeollabuk-do Province), which is the southernmost population in Korea. Of the six accessions, four *H. capitata* accessions from Japan were included for the first time in the phylogenomic and comparative analysis of *Hosta*. These four accessions were sampled from Kochi Prefecture (Shikoku), Shiraiwa Mountain (Miyazaki Prefecture, Kyushu), Rokko Mountain (Hyogo Prefecture, Honshu), and the Iya Valley (Tokushima Prefecture, Honshu). The accession from Iya Valley represents the type locality of *H. capitata*. For the Korean endemic *H. jonesii*, we sampled one accession from the type locality (GeumSan Mountain, Gyeongsangnam-do Province). One more Korean endemic, *H. yingeri*, was sampled from Hongdo Island, representing one additional accession near the type locality, Daeheuksando Island. These two additional accessions of Korean endemics were included in determining infraspecific variation within each taxon. In Japan, two endemic *Hosta* species, *H. tibae* and *H. tsushimensis*, were sampled from Iwaya Mountain (Nagasaki Prefecture, Kyushu) and Tsushima City (Nagasaki Prefecture, Kyushu), respectively. Lastly, the widely distributed Japanese endemic *H. sieboldiana*, a representative of subg. *Giboshi*, sect. *Helipteroides* was sampled from Girinsan Mountain (Niigata Prefecture, Honshu). While the current sampling strategy may not be sufficient to cover *Hosta* species in Japan, this taxon should give us some characteristics of plastomes in subg. *Giboshi*. *Hosta laevigata*, a narrow endemic to Heuksando Island, Korea, was not included in this study because it has not been found in its type locality, Heuksando Island, and is considered a hybrid [4,12]. Together, this study included all samples from subg. *Hosta* (*H. plantaginea*), three sections of subg. *Bryocles* (sect. *Bryocles*, *H. ventricosa*; sect. *Lamellatae*, *H. venusta*, *H. minor*, *H. jonesii*, *H. tibae*, and *H. tsushimensis*; sect. *Stoloniferae*, *H. clausa*, *H. capitata*, and *H. yingeri*), and one section *of Helipteroides* of subg. *Giboshi* [4]. All voucher specimens were deposited at the Changwon National University Herbarium.

### 4.2. DNA Extraction, Sequencing, Plastome Assembly, and Annotation

Fresh leaves were collected and dried using silica gel before DNA extraction. Total DNA was extracted using a DNeasy Plant Mini Kit (Qiagen, Carlsbad, CA, USA) and sequenced using an Illumina HiSeq 4000 sequencer (Illumina, Inc., San Diego, CA, USA), yielding 150 bp paired-end read lengths at Macrogen Corp. (Seoul, Korea). The resulting paired-end reads were assembled de novo using Velvet v1.2.10 with multiple k-mers [62] at coverage from 149 to 755. The tRNAs were confirmed using tRNAscan-SE [63]. Sequences were annotated using Geneious R10 [64] and deposited in GenBank (*H. capitata* from Mt. BaekUn, MZ919305; *H. capitata* from Mt. DaeDuck, MZ919306; *H. capitata* from Iya valley, MZ919307; *H. capitata* from Mt. Rokko, MZ919308; *H. capitata* from Pref. Kochi, MZ919309; *H. capitata* from Pref. Miyazaki, MZ919310; *H. jonesii*, MZ919311; *H. sieboldiana*, MZ919312; *H. tibae*, MZ919313; *H. tsushimensis*, MZ919314 and *H. yingeri*, MZ919315). Annotated sequence files in the GenBank format were used to draw a circular map with OGDRAW v1.3.1 [65].

### 4.3. Comparative Plastome Analysis

Using the Shuffle-LAGAN mode [66] of mVISTA [67], 12 complete plastomes of *Hosta* species representing different subgenera (*Bryocles*, *Giboshi*, and *Hosta*) and sections (*Lamellatae*, *Stoloniferae*, and *Bryocles*) were compared, including the previously published plastome of *H. clausa* (NC046896). A total of 12 *Hosta* plastomes, including the previously reported *H. minor* (MK732316), *H. plantaginea* (MT810382), and *H. venusta* (MK732314), were aligned using the back-translation approach with MAFFT ver. 7 [68] and manually edited with Geneious R10 [64]. Using DnaSP 6.10 [69], sliding window analysis with a step size of 200 bp and window length of 800 bp was performed to determine the nucleotide diversity (Pi, π) of the plastomes. Codon usage frequency was calculated using MEGA 7 [70] based on the RSCU value [71], which is a simple measure of non-uniform usage of synonymous codons in a coding sequence. The DNA code used by bacteria, archaea, prokaryotic viruses, and chloroplast proteins was used [72]. Protein-coding genes were run using the PREP suite [73] with 35 reference genes and a cut-off value of 0.8 to predict possible RNA editing sites in 12 *Hosta* plastomes. Analyses based on complete plastomes and concatenated sequences of 77 common protein-coding genes of the studied *Hosta* species were performed using MAFFT ver. 7 [68] in Geneious R10 [64]. To evaluate the natural selection pressure on the protein-coding genes of 12 *Hosta* plastomes, a site-specific model was tested using EasyCodeML [33] with the CODEML algorithm [34]. Seven codon substitution models (M0, M1a, M2a, M3, M7, M8, and M8a) were compared to detect positively selected sites based on the likelihood ratio test (LRT). Lastly, the pairwise sequence distance within and between species was calculated by the Kimura 2-parameter (K2P) method [74] using PAUP* 4.0a [75].

### 4.4. Phylogenetic Analysis

For phylogenetic analysis, complete plastome sequences of 21 accessions of *Hosta* and sequences of two *Yucca* plastomes were aligned with MAFFT ver. 7 [68] in Geneious R10 [64]. Twenty-one *Hosta* accessions included previously published ones, such as *H. clausa* (NC046896), *H. clausa* var. *ensata* (MN901630), *H. capitata* (NC045519), *H. jonesii* (NC046897), *H. minor* (MK732316 & NC035999), *H. plantaginea* (MT810382), *H. ventricosa* (NC032706), *H. venusta* (NC046895), *H. yingeri* (NC039976) and 11 newly sequenced plastomes in this study (six accessions of *H. capitata*, one accession of *H. jonesii*, *H. sieboldiana*, *H. yingeri*, *H. tibae*, and *H. tsushimensis*). Two *Yucca* species, *Y. brevifolia* (NC032711) and *Y. schidigera* (NC032714), were used as outgroups based on a previous study [24]. In addition to the analysis based on complete plastome sequences, we also performed a phylogenetic analysis based on concatenated protein-coding gene sequences. ML analysis based on the best-fit model of “K3Pu+F+I” was conducted using IQ-TREE 1.4.2 [76]. A non-parametric bootstrap analysis was performed with 1000 replicates.

## Figures and Tables

**Table 1:** Summary of the characteristics of the 11 *Hosta* chloroplast genomes.

Taxa	*Hosta* *capitata* (Mt. BaekUn)	*Hosta* *capitata* (Mt. DaeDuck)	*Hosta* *capitata* (Iya Valley)	*Hosta* *capitata* (Mt. Rokko)	*Hosta* *capitata* (Pref. Kochi)	*Hosta* *capitata* (Pref. Miyazaki)	*Hosta* *jonesii*	*Hosta* *sieboldiana*	*Hosta* *tibae*	*Hosta* *tsushimensis*	*Hosta* *yingeri*
Total cpDNA size (bp)	156,419	156,423	156,682	156,678	156,675	156,685	156,724	156,576	156,626	156,714	156,755
GC content (%)	37.82	37.82	37.81	37.81	37.81	37.81	37.81	37.81	37.82	37.8	37.8
LSC size (bp) /GC content (%)	84,785 /35.89	84,795 /35.88	85,054 /35.87	85,054 /35.87	85,049 /35.87	85,054 /35.87	85,104 /35.88	84,952 /35.87	85,014 /35.9	85,100 /35.86	85,115 /35.88
IR size (bp) /GC content (%)	26,711 /42.91	26,711 /42.91	26,711 /42.91	26,711 /42.91	26,711 /42.91	26,711 /42.91	26,698 /42.93	26,696 /42.93	26,697 /42.92	26,698 /42.92	26,704 /42.91
SSC size (bp) /GC content (%)	18,205 /31.9	18,206 /31.9	18,206 /31.91	18,202 /31.91	18,204 /31.91	18,209 /31.89	18,224 /31.82	18,232 /31.85	18,218 /31.87	18,218 /31.87	18,232 /31.86
Accession number	MZ919305	MZ919306	MZ919307	MZ919308	MZ919309	MZ919310	MZ919311	MZ919312	MZ919313	MZ919314	MZ919315

LSC, large single-copy region; IR, inverted repeat; SSC, small single-copy region.

**Table 2:** Log-likelihood values of the site-specific models, with detected sites having dN/dS values >1.

Gene Name	Models	np	ln L	Likelihood Ratio Test *p*-Value	Positively Selected Sites
*rbcL*	M8	27	−2050.484207	<0.000000001	477 Q 0.973 *
	M7	25	−2092.011742		
*rpoB*	M8	27	−4391.457170	<0.000000001	514 N 0.962 *
	M7	25	−4436.574724		
*rpoC2*	M8	27	−5749.647291	<0.000000001	754 W 0.989 *
	M7	25	−5784.027018		
*rpl16*	M8	27	−565.310154	0.000009183	5 K 0.966 *; 42 I 0.966 *
	M7	25	−576.908351		
*rpl20*	M8	27	−531.912203	<0.000000001	76 H 0.998 **; 80 G 0.980 *
	M7	25	−574.476753		

* *p* < 0.05; ** *p* < 0.01. np represents degrees of freedom.

## Data Availability

The chloroplast sequence data presented in this study are available in GenBank under the accession numbers MZ919305-MZ919315. The raw sequenced NGS data are available in the National Center for Biotechnology Information (NCBI) Sequence Read Archive (BIOProject number PRJNA758128; SRA accession numbers SRR15652493, and SRR16554751-15664760).

## Supplementary Materials

The following are available online at https://www.mdpi.com/article/10.3390/plants10101980/s1, Table S1: Codon usage and the codon-anticodon recognition pattern for tRNA in 12 *Hosta* plastomes, Table S2: The predicted RNA editing sites in the complete chloroplast genome of 12 *Hosta* species, Table S3: The pairwise sequence distance (K2P) within and between *Hosta* species, Table S4: List of samples for the complete chloroplast genome of *Hosta* in Japan and Korea.
